# Supplementary material for: Process Evaluation of a Comprehensive Intervention for the Early Detection and Prevention of Oral Cancer: A Mixed Methods Study
Source: Int J Environ Res Public Health. 2022 Jun 10;19(12):7120. doi: 10.3390/ijerph19127120 (PMC9223362; doi:10.3390/ijerph19127120)
Supplement: Supplementary file 1 [file ijerph-19-07120-s001.zip › ijerph-1747867-supplementary.pdf]

Journal of Cancer Education
https://doi.org/10.1007/s13187-022-02146-y

Using Intervention Mapping to Develop ISAC, a Comprehensive Intervention for Early Detection and Prevention of Oral Cancer in Saudi Arabia

Mohammed Jafer[1,2] · Ibtisam Moafa[1,2] · Rik Crutzen[1] · Bart van den Borne[2]

Accepted: 6 February 2022
© The Author(s) 2022

Abstract

Oral cancer forms a major public health issue. In Saudi Arabia, Jazan region has the highest rate of oral cancer; > 35% of total cases. Furthermore, dentists' engagement in oral cancer screening and patient education in Jazan region is limited. This paper aimed to describe the process used to develop a comprehensive oral cancer (OC) practice intervention to be implemented in dental clinics. The intervention was informed by the six steps of intervention mapping (IM). Steps 1–3 included mixed methods approach of reviewing relevant existing literature, focus group discussions, observations, one-on-one interviews, and questionnaires utilizing the community participatory approach. Step 4 used information form steps 1–3 to develop the intervention components and its associated tools to facilitate its delivery. Steps 5 and 6 specified the prospective plans for implementation and evaluation. ISAC is the developed intervention that comprises the following: *Informing* dental patients about performing routine OC screenings, *Screenings* for OC, *Advising* patients, and *Connecting* patients to the required services. ISAC practical applications were clustered into two components: (a) didactical session covering aspects related to OC practices and introducing ISAC and (b) practical session that included a step-by-step modeling of the intervention. Using IM facilitated the systematic planning of the ISAC intervention that covers the main issues revealed by the need's assessments. Working towards developing the ISAC required extensive work in assessing dental public health issues in a specific context with limited data — and this constituted a great challenge. The development of the ISAC was a lesson that casts light on the advantages of engaging multidisciplinary expertise to tackle serious public health issue like OC.

Keywords Oral cancer · Intervention mapping · Methods · Program development · Prevention · Early detection · Dental practice · Oral health · Behavior change

Background

Oral cancer (OC), and particularly squamous cell carcinoma, is one of the most prevalent cancers in Saudi Arabia [1]. In comparison to other regions of Saudi Arabia, the Jazan region carried the heaviest burden of the disease, as it had more than 35% of the registered diagnosed cases at an advanced stage, with a slightly higher rate in females (compared to men) [1]. A strong association was found between these diagnosed cases and the use of smokeless tobacco [2]. The form of smokeless tobacco that is commonly used in the Jazan region is known as Shammah. Despite the disease being so prevalent in Saudi Arabia, and particularly in the Jazan region, there has been limited effort to prevent its risk factors or to screen for it in clinics [3]. Evidence had shown that oral cancer can be prevented by eliminating its risk factors [4]. Moreover, it was found that early detection of OC can lead to a better prognosis for the recovery from the disease [5]. Due to this, dentists screening for OC and educating patients on its risk factors is highly recommended [6]. However, these recommendations are not usually met by dentists [7]. Similarly, in the Jazan region, dentists' engagement in OC screening and patient education has been found to be limited [3]. Several factors were suggested as contributing factors towards this kind of behavior

✉ Mohammed Jafer
m.jafer@maastrichtuniversity.nl

1 Dental Public Health, Department of Preventive Dental Science, College of Dentistry, Jazan University, Jazan, Saudi Arabia

2 Department of Health Promotion, Maastricht University/CAPHRI, P.O. Box 616, Maastricht 6200 MD, The Netherlands

Published online: 11 February 2022

by dentists — for example, limited exposure to OC cases, a lack of OC screening skills, and a lack of skill in educating patients [3]. Therefore, it is essential to have an intervention that aims to improve OC practices in the Jazan region. The objective of this paper was to report on the development of a comprehensive intervention for OC practices (ISAC), using the Intervention Mapping approach [8].

Methods

We believe that Intervention Mapping (IM) was the most appropriate approach because it addresses this challenge from different perspectives. [8]. IM is a systematic framework that is characterized by three aspects: (1) application of a social ecological model that views the individual behavior as an outcome of the interaction of the individual with physical, social, and organization environments; (2) community broad participation to enhance the relevance, acceptability, and cultural suitability; (3) utilizes theory and evidence as foundations in order to assess and develop effective interventions for behavior and environmental changes that are conducive to health. IM entails six steps: (1) Logic model of problem; (2) logic model of change; (3) program design; (4) program production; (5) implementation plan; and (6) evaluation plan [8]. Each of these steps involves several tasks and build in an iterative and a cumulative process [8]. The research team with representation from dental public health, patients' education, behavior change, and technology have established the planning group. This planning group included all potential intervention implementers of essential value — including the Dean and the Head of the Community Dentistry Division (CDD) of Jazan Dental School (JDS) [9]. Moreover, a bottom-up community empowerment approach sought to include the target group: dentists, dental interns, and dental patients of JDS (from both male and female branches) to create a compatible intervention that ensured dental best practices and ultimately improve patient quality of life [8, 10]. All the research team members are experienced with Intervention Mapping (IM). The team has followed the six steps of IM for the development, implementation, and evaluation of the intervention. This paper describes the process of intervention development, intervention protocol for implementation, and evaluation in steps 1 to 6 of IM.

Step 1: Logic Model of the Problem We have conducted needs assessments that involved the following: (a) Evidence reviews of existing data on OC problem at Jazan region; (b) a quantitative study to assess dentists' knowledge of OC [11]; a qualitative study to explore dentists' perspectives related to OC utilizing the grounded theory approach [12]; (c) direct clinical observation of dentists in their routine dental practices by four calibrated dentists (the observers who received calibration trainings to improve their scoring accuracy and consistency) [13]; (d) an exploratory sequential mixed methods design to investigate dental patients' behavior, thoughts, opinions, and needs for oral cancer information, and dentists' behavior regarding prevention and examination of oral cancer [14]; and (e) an assessment and evaluation of JDS organization context factors [12–14].

Step 2: Program Outcomes, Objectives, and Logic Model of Change We have utilized the findings from step 1 to determine who and what needs to be changed in order to increase the early detection and prevention of OC. We've formulated the desired outcomes using SMART objectives, which stands for Specific, Measurable, Achievable, Realistic, and Time, and we finally created the logic model of change.

Steps 3 and 4: Program design and Program Production We have defined the intervention theme, components, scope, and sequence. Additionally, we have identified the behavioral change methods targeting the relevant determinants. These determinants were translated into practical applications considering their parameters for use to optimize effectiveness. Aside from this, the intervention channel/vehicle, materials, length, quality, and feasibility were distinguished for each component. Finally, the intervention material resources were prepared in collaboration with experts in oral cancer, digital design, health education, and promotion.

Steps 5 and 6: Program Implementation and Evaluation Plan We have written the evaluation questions (process/effect) considering the intervention logic models, goals, objectives, and matrices. Effect evaluation investigates whether the behavior, environmental outcomes, and objectives change because of the intervention [8, 15], while the process evaluation assesses the intervention implementation and delivery [8]. Following this, indicators and measures were determined to assess the formulated questions on the effect and process evaluation. Finally, we have specified the methodological design for conducting process and effect evaluation.

Results

The results of the needs assessment revealed the following:

Knowledge [11] A total of 237 individuals participated (72 students, 68 dental interns, and 88 faculty members of different nationalities) in which 55.1% were males and 44.9% were females. The average knowledge of OC and its risk factors among last year students, interns, and faculty members was at a moderate level; 20.2 ± 3.6 out of 35. The questions

regarding the risk factors of OC among females in particular were answered correctly by only 28% of dentists. Majority of the participants had a high level of knowledge about how to preform OC examination but a low level of knowledge regarding the sites and clinical manifestation of the disease as well as it epidemiology.

Dentists' Perceptions Toward OC [12] FGDs revealed the following themes representing participants' thoughts about OC: (1) OC in Jazan region as a public health issue; (2) behavioral and cultural related risk factors attributed to tobacco consumption; (3) impact of JDS curriculum on OC recent and future dental practice; (4) clinicians' behavior toward OC; and (5) challenges and barriers toward OC clinical practice.

Direct Clinical Observation [13] Ninety-five examiners (final-year students, dental interns, and faculty members) and 32 patients participated in the study. A total of 70% of examiners investigated the systemic diseases and < 30% investigated tobacco use and oral hygiene practices. A total of 90% of the examiners assessed patients' dentations and < 50% assessed lymph nodes of the neck, lip, check, tongue, palate, or floor of the mouth. Only three female final-year dental students had requested specialist consultations, as well as only 11 provided advice to the patients. A significant difference between examiner groups was found in favor of faculty members ($p = 0.007$ 95% *CI*: 3.08–23.53). Twenty-three participants participated in the two follow-up FGDs to discuss the factors possibly associated with the observed items' scores. Dependence on previous dental examination was elicited to be generally related to the low-score items in the checklist. Other factors included lack of confidence to identify oral precancerous/cancerous lesion, to provide tailored risk factor education or to provide tobacco counseling as they lacked formal training on these skills. Participants linked the cultural and religious unacceptability of alcohol use to the observed low score in asking about it. For items related to tobacco and advice on OC risk factors, female students and interns had higher scores than males and it was justified as related to the fact that female students/interns are vigilant to the oral changes associated with tobacco as they are used to examine mainly female patients who are usually non-smokers. However, female participants had given tobacco advice to the patients based on their personal beliefs as they did not receive formal training on tobacco cessation. Dental interns revealed two factors related to their general low score in comparison to students and faculty members: they rely on the other dentists whom the patient will be referred to in the next appointment, and because they have a busy clinical schedule with a high number of patients, and therefore they cannot perform full oral screening on each patient.

Dental Patients' Perceptions and Needs Concerning OC Information, Examination, Prevention and Behavior [14] The qualitative analysis of interviews showed three major themes: knowledge regarding OC and its associated aspects, perception of OC and its related aspects, and patients' behavior and their dentists' behavior regarding OC self-examination and clinical procedures. Several participants indicated that they had no idea of what oral cancer could mean and other participants thought that OC could be the result of some type of bacterial or fungal infection. Most of participants did not know the risk factors of OC. Several participants were not aware of the preventive measures they could take to avoid OC. Other participants thought that regular dental check-ups could prevent oral cancer.

The follow-up quantitative study included 315 patients. The mean participant age was 31 ± 11 years (range of 12–70). Among the 313 participants who reported their gender, 41.2% were males and 58.8% were females. Majority were Saudis (85.9%). Participants reported their levels of education as follows: 4.4% were uneducated, 7.9% had primary education, 15.6% had intermediate education, 24.1% had secondary education, and 47.2% had university education. The study findings revealed that patients' OC knowledge levels were adequate, but most reported that their dentist had never examined them for OC. Furthermore, they had never performed self-examinations for OC, nor were they aware of the possibility of doing so. Participants showed a preference for being examined and educated by their dentist about oral cancer and believed it would help early detection. Patients felt a need for more attention to be paid to OC examinations, preventive measures, and targeted information on OC risk factors.

Key Findings of Steps 1 and 2 The findings from the needs' assessments in addition to group brainstorming sessions revealed a gap that exists between knowledge and practice of OC examination among JDS dentists [3, 11–14]. The main determinants that were found to be related to the personal contributing behaviors were as follows: low awareness of OC status in Jazan, dentists' lack of experience, skills and self-efficacy, and the negative descriptive norms regarding oral cancer practices in JDS [3, 11–13]. The determinants of the environmental behaviors were interns lacking exposure to OC patients and having clinical guidelines that do not include OC. The logic model of the problem shows a detailed description of the OC problem and the relationships between the factors associated with it (See Fig. 1).

The agreed expected outcomes were as follows: (a) all dental interns performing complete OC practices (examination and patient education) at JDS clinics within 1 year of implementation and (b) Clinical Director including the complete OC practice in the clinical guidelines and adding

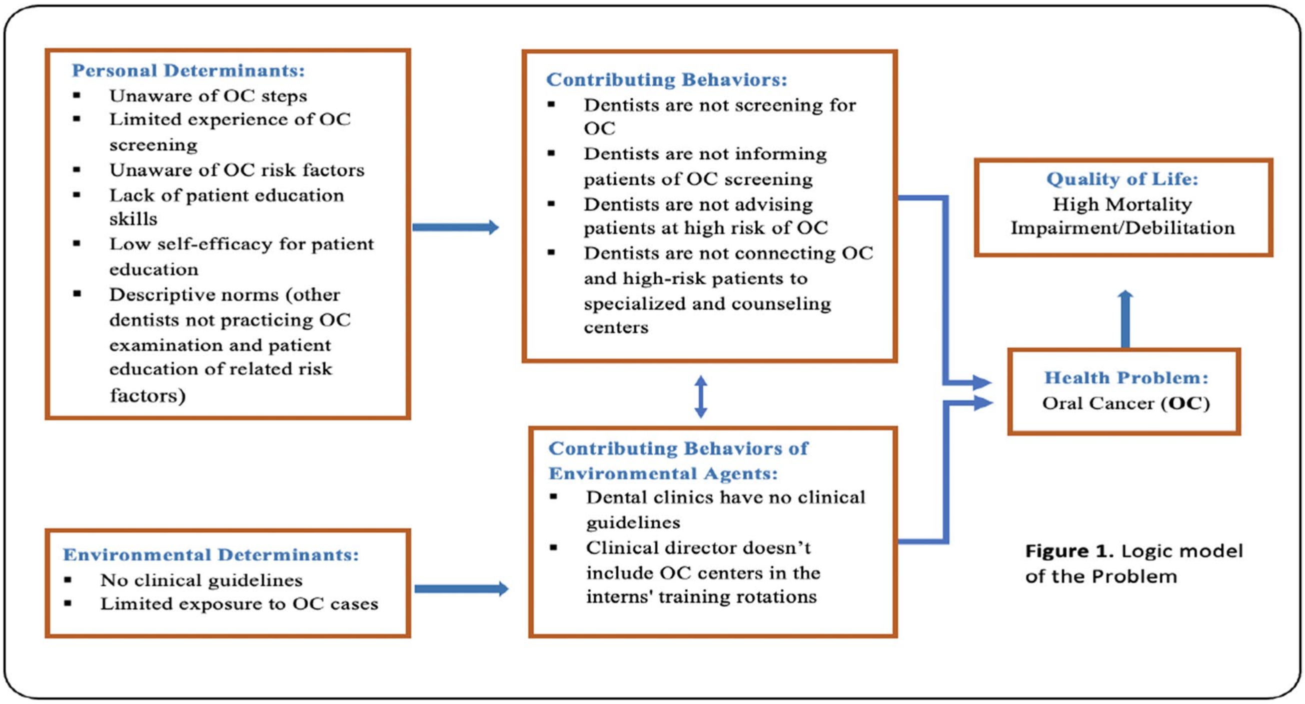

Fig. 1 Logic model of the problem

OC centers to the interns' training schedule at JDS clinics within 1 year of implementation.

Dental interns were the target group because they are the first to see the patients in JDS-clinics and they had the lowest score in terms of performing OC examination and patient education [13]. The intervention period was specified as 1 year due to the structure of JDS interns' rotations, as all interns must practice at JDS within their 1-year internship. The clinical director was chosen because he is the main person in charge of internships in JDS. After formulating the outcome, we have specified the performance objectives for behavioral and environmental agents which are the exact actions needed to be carried out by individuals to achieve the behavioral change outcome [8]. The performance objectives for dental interns included the following: (a) dentists inform their patients of OC screening; (b) dentists perform full OC screenings on their patients; (c) dentists advising their OC and high-risk patients; and (d) dentists connecting their OC and high-risk patients with specialized clinics and counseling centers. While the performance objectives of the clinical director included the following: (a) Clinical Director includes OC practices, e.g., ISAC into the clinical guidelines and (b) Clinical Director increases interns' exposure to OC patients by adding OC centers to their interns' training rotations.

Subsequently, the matrices of change objectives were formulated which symbolize the pathways for the most immediate changes in the targeted determinants, which influence the individual and environmental agent's behavior [8]. Based on current literature, experiences and findings from needs assessments, the main behavioral determinants that need to be modified to achieve the performance objectives for dental interns, were awareness, skills, self-efficacy, and descriptive norms. These determinants were evaluated according to its importance and changeability in literature. A detailed description of the change objectives' matrices and determinants of change is accessible on https://osf.io/epnwx/. Finally, the logic model of change was constructed to illustrate the potential relations between theory and evidence-based methods, influencing determinants, and behavioral and environmental outcome (See Fig. 2).

The Intervention ISAC was determined as the intervention theme. ISAC is an acronym for a new evidence-based intervention for comprehensive OC dental practices, which stands for $I = Inform$ (verbally and documentation): dental interns will inform their patients about performing OC examinations as part of the routine dental examination practice and include the action of informing of this in the clinical examination documentation; $S = Screen$, with two main parts: the first part is taking medical history, according to the clinical guidelines as well as including asking about the local risk factors, such as smokeless tobacco use and water-pipe smoking. The second part is a clinical examination according to the clinical guidelines, which includes

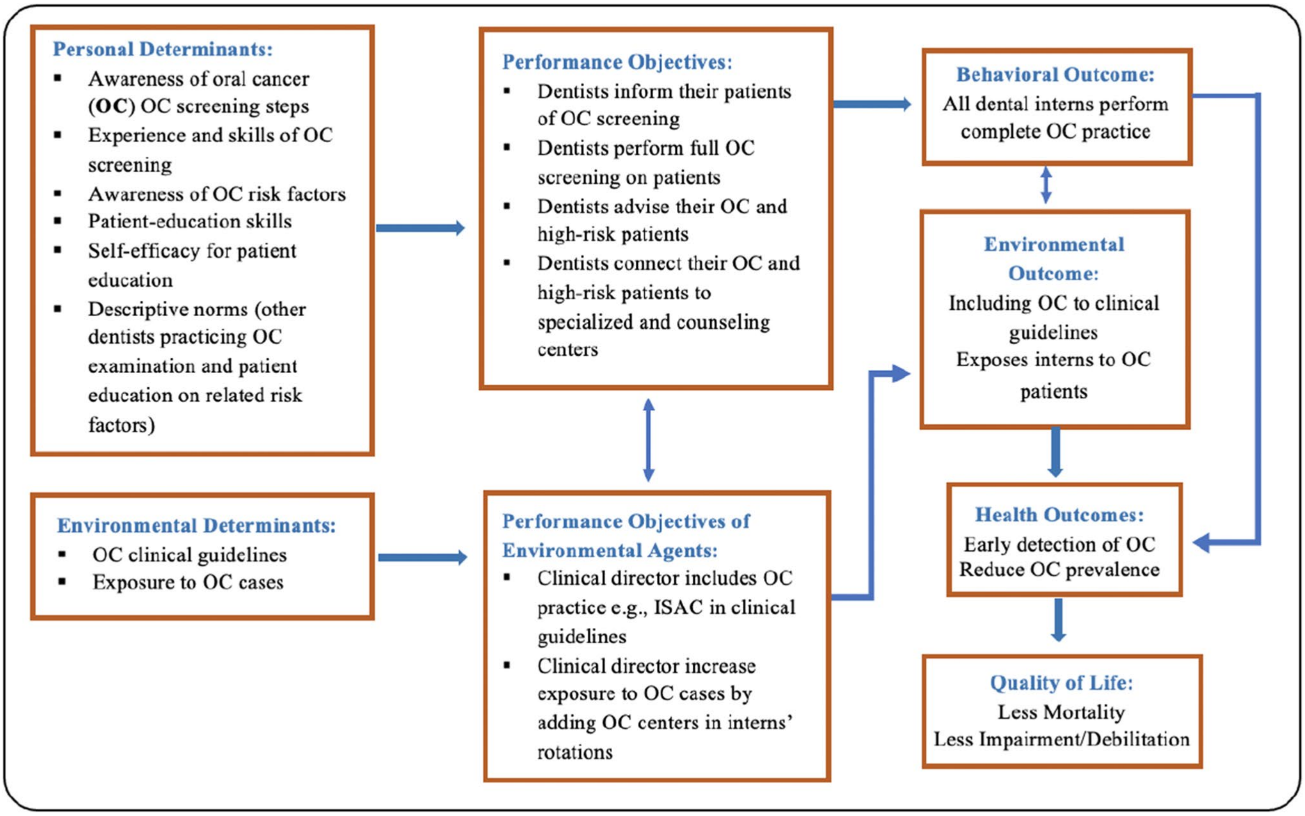

Fig. 2 Logic model of change

screening for OC; $A = $ *Advice*: patients at high risk (e.g., users of smokeless tobacco) will be counseled to aid cessation, using clear and tailored language to deliver health messages; and $C = $ *Connect*, with two dimensions: the first dimension is to connect the patient that has any suspicious lesions with specialized centers that are qualified in dealing with OC cases, such as Prince Mohammed Bin Nasser Hospital (PMBN) in the Jazan region. The second dimension is to connect tobacco users with a designated service to stop using tobacco products.

Potential Adopters and Implementers The potential adopter of ISAC intervention in JDS is the clinical director. The implementers of ISAC will be the Community Dentistry Division (CDD). The expected outcome of the implementation plan is as follows: All faculty members in CDD at JDS will implement ISAC with high fidelity and completeness within 1 year. In order to reach the performance objectives, certain determinants were identified and evaluated according to their importance and changeability in the literature [15, 16]: knowledge, attitude, self-efficacy, and skills toward ISAC.

ISAI Intervention Delivery, Implementation and Evaluation ISAC will be delivered as a workshop that targets JDS dental interns and consists of didactic and practical components. Table 1 provides a detailed description of each component. The selected theory and evidence-based behavior change methods for dental interns and the Clinical Director were as follows: *consciousness raising, guided practice, information on the approval of others,* and *persuasive communication* (Table 1). Additional information is accessible on < https://osf.io/6g9pd/ >. ISAC intervention components and materials will be pre-tested using thinking-aloud, expert evaluation, and questionnaire piloting, in order to optimize the content and execution. The objectives of the pre-test were based on the change method parameters < https://osf.io/j9e28/ >, to test the concept (dental interns, CDD), readability (dental interns), message execution (dental interns), and the implementation factors to determine the perceptions of the Clinical Director and the CDD of ISAC's complexity, trialability, relative advantage, and to predict possible problems with implementation < https://osf.io/fapc3/ > [9].

The effect-evaluation questions on health, quality of life, behavior, and environment as well as the methodological design for conducting the effect and the process evaluations are described in detail in < https://osf.io/38dy6/ >.

Table 1 Change objective for dental interns — methods and application

Determinants and change objective		Methods	Parameters	Applications for JDS	Channel for JDS
Awareness	Acknowledge the importance of communicating with patients<br>Acknowledge the importance of full oral cancer screening<br>Acknowledge the importance of advising their patients<br>List the specialized centers and counseling services	Consciousness raising	Feedback and confrontation; raising awareness must be followed by self-efficacy	The trainer gives a lecture addressing: general/local oral cancer epidemiology, general/local oral cancer risk factors and their affects, full oral cancer screenings, the importance of patient education and its applications, introducing the ISAC method, introducing tobacco-cessation services	Lecture
Self-efficacy and Skills	Express confidence and demonstrate ability in informing their patients of oral cancer screening<br>Express confidence and demonstrate ability in performing full oral cancer screening<br>Express confidence and demonstrate ability in advising their patients regarding their oral cancer status or regarding oral cancer risk factors<br>Express confidence and demonstrate ability in referring oral cancer patients	Guided Practice (reinforcement, vicarious learning)	Subskill demonstration, Instruction, and enactment with individual feedback; requires supervision by an experienced person	First: interns have a session with trainer who models practice ISAC<br>Second: interns engage in a role-play where they apply ISAC in two groups; practicing and observing<br>Third: they give feedback to each other under supervision of trainer and receive positive comments from the supervisors and each other<br>Fourth: workshop certificate signed by the JDS Dean	Group communication and mutual support
Descriptive norms	Recognize that other dentists inform their patients of oral cancer<br>Recognize that other dentists perform full oral cancer screenings for their patients<br>Recognize that other dentists advise their patients regarding their oral cancer status or regarding oral cancer risk factors<br>Recognize that other dentists connect their patients to tobacco-quitting services	Information about others' approval	Positive expectations are available in the environment	Informing interns that all JDS clinicians will be engaging in ISAC practice	In both lectures, group communication and mutual support

Discussion

In Jazan, where the prevalence of OC and its risk factors is high [2], there are urgent efforts needed to control the disease in the region. There had only been limited attempts to raise awareness of OC by a few dental-students' volunteers [3]. Therefore, the research team took the initiative to challenge OC burden and its risk factors in the region from different approaches. One of these approaches was through utilizing intervention mapping, which engaged stakeholders, oral healthcare providers, and citizens in Jazan. As a result, the current developed intervention (ISAC) aimed to improve OC practices at Jazan University by preparing its dental graduates to perform full OC clinical examinations and patient education, which will lead to early detection and prevention of OC.

The needs assessment, at both the level of dentists and patients, has revealed major issues relating to dentists' behavior toward OC practice and educating patients — for example, not informing patients of OC examinations, not performing full OC examinations, and not educating their patients on the risk factors, if needed, or referring them to tobacco-cessation services [3, 11, 13]. Those major issues were covered in the main pillars of ISAC. Furthermore, upon assessing the contextual characteristics of JDS, several factors were found to be positive in the progress toward implementing the interventions. An interesting example of one of these factors was the implementation climate (dentists' shared perceptions of the importance of intervention implementation within the JDS that results from dentists' shared experiences, observations of and their information about JDS implementation policies) which will provide a supportive context for implementing ISAC. The effect of this strategic climate is believed to be most proximal to the effective implementation of the intervention [17, 18]. Moreover, it reflects oral healthcare providers' perceptions toward the priorities of JDS, from what they learned as a shared assumption from JDS policies, procedures, and communications (formal and informal) with JDS leaders [18, 19].

It should be noted that patients' engagement in the present protocol was not tokenistic participation, but rather, patients had a significant contribution to the process of developing the intervention protocol. In addition to dentists' (faculty member, intern, and students) role, patients' knowledge, opinions, perceptions, and practice regarding OC and OC examinations have added significant input to the intervention development as it brought innovative insights toward OC practices in the region of Jazan. Furthermore, patients provided an indirect objective assessment for dentists' practice of OC examinations and patient education based on patients' real experience which enhanced the credibility of the findings [20]. Moreover, patients' opinions and advice regarding the best method and approach to enhance the OC examinations practice in Jazan region and the possible facilitators and barriers toward OC interventions were incorporated in the intervention design. However, as described in our evaluation plan summary, the dissemination of the intervention findings will target relevant stakeholders including research team, scientific literature, JDS, policymakers, health promoters and dental schools in areas that share the similar burden of oral cancer high rate, e.g., Sudan and Yemen.

Conclusions

Using IM facilitated in planning and designing the current developed intervention for the early detection and prevention of OC. Working towards developing ISAC required extensive work in assessing dental public health issues in a specific context with limited data — and this constituted a great challenge. However, it enriched the research team's understanding of OC in the Jazan region and its local risk factors — for example, the use of Shammah — through investing effort to investigate the issue from different angles, including its clinical practice by oral healthcare providers. Furthermore, the development of ISAC was, in itself, a lesson that casts light on the advantages of engaging multidisciplinary expertise to tackle a dental public health issue like OC. These advantages can be seen clearly when deciding what behavioral and environmental determinants need to be targeted and when weighing these determinants based on their importance and changeability using theoretical and empirical evidence. In addition, the fact that ISAC was a response to JDS administration's request to develop an intervention that targets OC and its related health risk behaviors would aid in its successful implementation [18].

Abbreviations OC: Oral cancer; IM: Intervention mapping; JDS: Jazan Dental School; CDD: Community Dentistry Division; ISAC: Inform, screen, advise and connect

Author Contribution Jafer M., Crutzen R. and Borne B. contributed to the design and the conception of the research. Jafer M. and Moafa I. were involved in data acquisition. All authors had substantial contribution in data analysis and interpretation as well as in manuscript preparation and drafting. Jafer M., Crutzen R., and Borne B have critically reviewed the manuscript. While the final version of the manuscript was approved for the publication by all authors. All authors contributed to the design and the conception of the protocol. MJ, IM, and BB are still contributing to the implementation of the intervention. All authors have critically reviewed the manuscript for intellectual content and have approved the final version of the manuscript for the publication.

Declarations

Ethics Approval The present intervention protocol was performed according to the ethical standards of the institutional research committee, as well as the 1964 Helsinki Declaration. The whole project received ethical approval from Jazan University; Registry no. [CDREC-06], dated 21 December 2016.

Consent to Participate Not Applicable.

Consent for Publication Not Applicable.

Conflicts of Interest The authors declare no competing interests.

Open Access This article is licensed under a Creative Commons Attribution 4.0 International License, which permits use, sharing, adaptation, distribution and reproduction in any medium or format, as long as you give appropriate credit to the original author(s) and the source, provide a link to the Creative Commons licence, and indicate if changes were made. The images or other third party material in this article are included in the article's Creative Commons licence, unless indicated otherwise in a credit line to the material. If material is not included in the article's Creative Commons licence and your intended use is not permitted by statutory regulation or exceeds the permitted use, you will need to obtain permission directly from the copyright holder. To view a copy of this licence, visit http://creativecommons.org/licenses/by/4.0/.

References

1. Saudi Cancer Registry. Saudi Arabia cancer incidence report 2012 [document on the Internet]. Riyadh, Saudi Arabia: Saudi Cancer Registry; 2015 [cited 2020 Apr 1]. Available from: http://ghdx.healthdata.org/organizations/saudi-cancer-registry
2. Allard WF, DeVol EB, Te OB (1999) Smokeless tobacco (Shamma) and oral cancer in Saudi Arabia. Community Dent Oral Epidemiol 27(6):398–405
3. Jafer M, Crutzen R, Moafa I, Van Den Borne B. What do dentists and dental-students think of oral cancer and its control and prevention strategies? A qualitative study in Jazan Dental School. J Canc Educ. 2019;134–142
4. Sankaranarayanan R, Ramadas K, Amarasinghe H, Subramanian S, Johnson N. Oral cancer: prevention, early detection, and treatment. In: Gelband H, Jha P, Sankaranarayanan R, Horton S, editors. Cancer: Disease Control Priorities [Internet]. 3rd ed (Volume 3). Washington (DC): The International Bank for Reconstruction and Development/The World Bank; 2015 [cited 2020 Apr 1]. Available from: https://www.ncbi.nlm.nih.gov/books/NBK343649/
5. Gomez I, Seoane J, Varela-Centelles P, Diz P, Takkouche B (2009) Is diagnostic delay related to advanced-stage oral cancer? A meta-analysis Eur J Oral Sci 117(5):541–546
6. Epstein JB, Huber MA (2015) The benefit and risk of screening for oral potentially malignant epithelial lesions and squamous cell carcinoma. Oral Surg Oral Med Oral Pathol Oral Radiol Endod 120(5):537–540
7. Warnakulasuriya KA, Johnson NW (1999) Dentists and oral cancer prevention in the UK: opinions, attitudes and practices to screening for mucosal lesions and to counselling patients on tobacco and alcohol use: baseline data from 1991. Oral Dis 5(1):10–14
8. Bartholomew Eldredge LK, Markham C, Ruiter R, Fernández M, Kok G, Parcel G (2016) Planning health promotion programs: an intervention mapping approach, 4th edn. Jossey-Bass & Pfeiffer Imprints, Wiley, San Francisco
9. Bonham CA, Sommerfeld D, Willging C, Aarons GA (2014) Organizational factors influencing implementation of evidence-based practices for integrated treatment in behavioral health agencies. Psychiatry J 2014:9. https://doi.org/10.1155/2014/802983
10. Minkler M, Wallerstein N, Wilson N. Improving health through community organization and community building. Health Educ Behav. 2008; 287–312.
11. Jafer M, Crutzen R, Jafer A, Van Den Borne B (2018) What do dental college clinicians know about oral cancer and its risk factors? An assessment among final year students, interns and faculty members in saudi arabia. J Clin Exp Dent 10(9):e908–e913. https://doi.org/10.4317/jced.55168
12. Jafer M, Crutzen R, Moafa I, van den Borne B (2021) What do dentists and dental students think of oral cancer and its control and prevention strategies? A qualitative study in Jazan Dental School. J Canc Educ 36(1):134–142
13. Jafer M, Crutzen R, Halboub E, Moafa I, Van Den Borne B, Bajonaid A (2020) Dentists behavioral factors influencing early detection of oral cancer: a direct clinical observational study. J Canc Educ. https://doi.org/10.1007/s13187-020-01903-1
14. Jafer M, Crutzen R, Ibrahim A, Moafa I, Zaylaee H, Ajeely M, van den Borne B, Zanza A, Testarelli L, Patil S (2021) Using the exploratory sequential mixed methods design to investigate dental patients' perceptions and needs concerning oral cancer information, examination, prevention and behavior. Int J Environ Res Public Health 18:7562. https://doi.org/10.3390/ijerph18147562
15. Fleuren M, Wiefferink K, Paulussen T (2004) Determinants of innovation within health care organizations: literature review and delphi study. Int J Qual Health Care 16(2):107–123
16. Basch CE (1984) Research on disseminating and implementing health education programs in schools. J Sch Health 54(6):57–66
17. Ehrhart MG, Aarons GA, Farahnak LR (2014) Assessing the organizational context for EBP implementation: the development and validity testing of the Implementation Climate Scale (ICS). Implement Sci 9(1):157
18. Chaudoir SR, Dugan AG, Barr CHI (2013) Measuring factors affecting implementation of health innovations: a systematic review of structural, organizational, provider, patient, and innovation level measures. Implement Sci 8:22. https://doi.org/10.1186/1748-5908-8-22
19. Judge TA, Piccolo RF (2004) Transformational and transactional leadership: a meta-analytic test of their relative validity. J Appl Psychol 89(5):755–768
20. Kimberlin CL, Winterstein AG (2008) Validity and reliability of measurement instruments used in research. Am J Health Syst Pharm 65(23):2276–2284

Publisher's Note Springer Nature remains neutral with regard to jurisdictional claims in published maps and institutional affiliations.
